# Supplementary material for: A mechanistic investigation of the Li10GeP2S12|LiNi1-x-yCoxMnyO2 interface stability in all-solid-state lithium batteries
Source: Nat Commun. 2021 Nov 18;12:6669. doi: 10.1038/s41467-021-26895-4 (PMC8602263; doi:10.1038/s41467-021-26895-4)
Supplement: Supplementary file 1 — Supplementary Information [file 41467_2021_26895_MOESM1_ESM.pdf]

Supplementary Information for

A mechanistic investigation of the $Li_{10}GeP_2S_{12}$|$LiNi_{1-x-y}Co_xMn_yO_2$ interface stability in all-solid-state lithium batteries

Tong-Tong Zuo[a,b,*], *Raffael Rueß*[a,b], *Ruijun Pan*[a,b,c], *Felix Walther*[a,b], *Marcus Rohnke*[a,b], *Satoshi Hori*[d], *Ryoji Kanno*[d], *Daniel Schröder*[e,*], *Jürgen Janek*[a,b,*]

[a] Institute of Physical Chemistry, Justus Liebig University Giessen, Heinrich-Buff-Ring 17, D-35392 Giessen, Germany

[b] Center for Materials Research (LaMa), Justus-Liebig-University Giessen, Heinrich-Buff-Ring 16, D-35392 Giessen, Germany

[c] Materials Science and Engineering Program & Texas Materials Institute, The University of Texas at Austin, Austin, Texas 78712, United States

[d] Institute of Innovative Research (IIR), All-Solid-State Battery Unit, Tokyo Institute of Technology, 4259 Nagatsuta, Midori-ku, Yokohama 226-8502, Japan

[e] Institute of Energy and Process Systems Engineering (InES), Technische Universität Braunschweig, 38106 Braunschweig, Germany

* corresponding authors:

tong-tong.zuo@pc.jlug.de; d.schroeder@tu-braunschweig.de; juergen.janek@pc.jlug.de

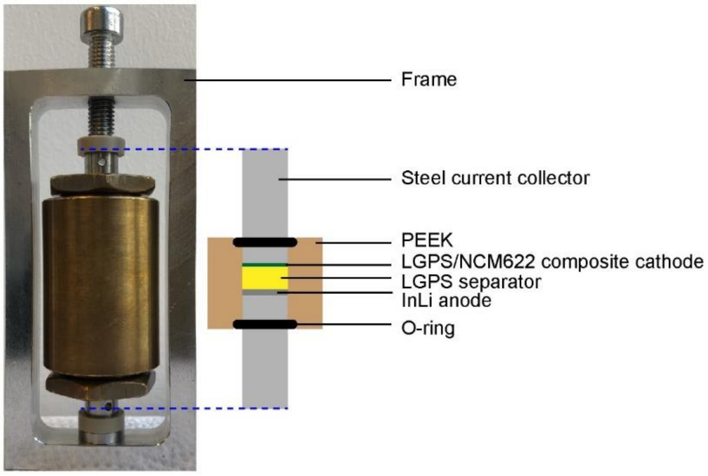

Supplementary Figure 1. The image of the homemade cell for electrochemical measurements. The aluminum frame was used to maintain the applied pressure.

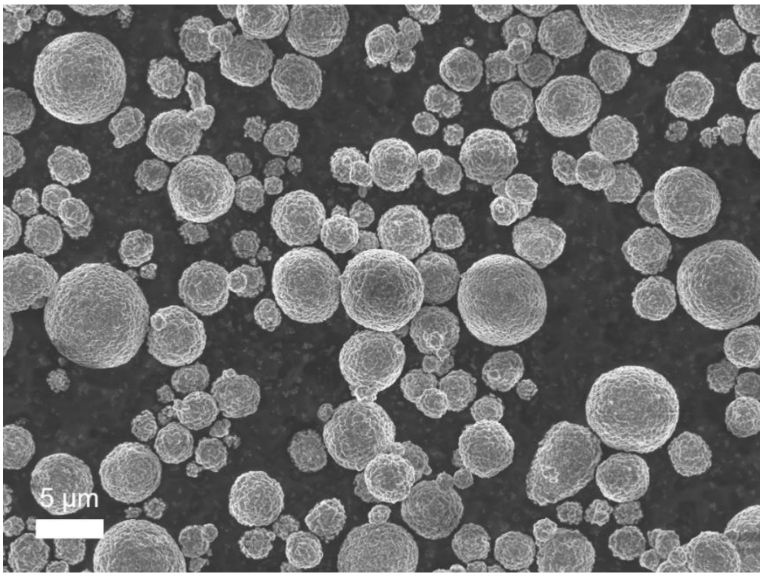

Supplementary Figure 2. SEM image NCM622 particles.

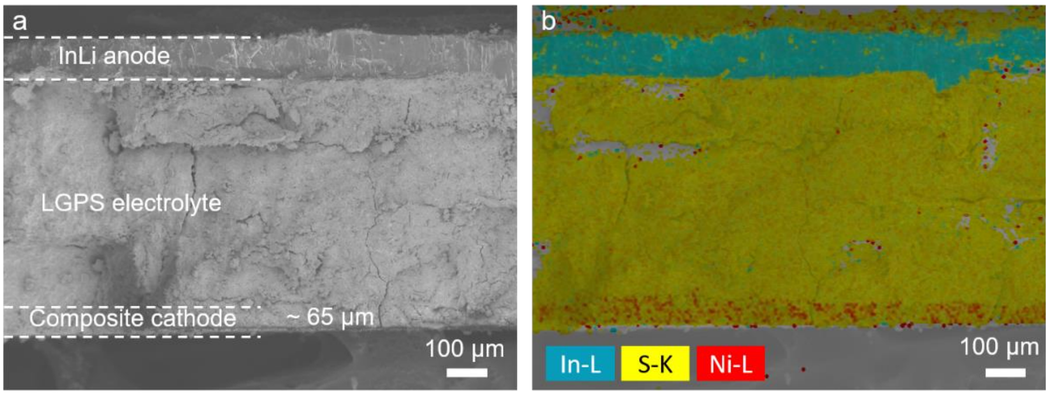

Supplementary Figure 3. (a) Cross-sectional SEM image and (b) the corresponding element mapping of the full cell.

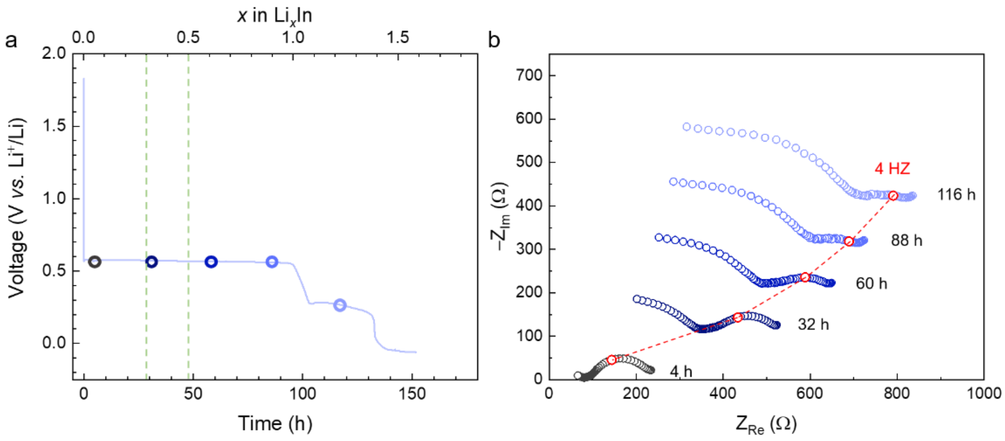

Supplementary Figure 4. (a) The voltage-time profile and (b) Nyquist plots of the Li|LGPS|In cell during lithiation process. The characteristic frequency (ca. 4Hz) of the LGPS|In/InLi interface was marked in (b).

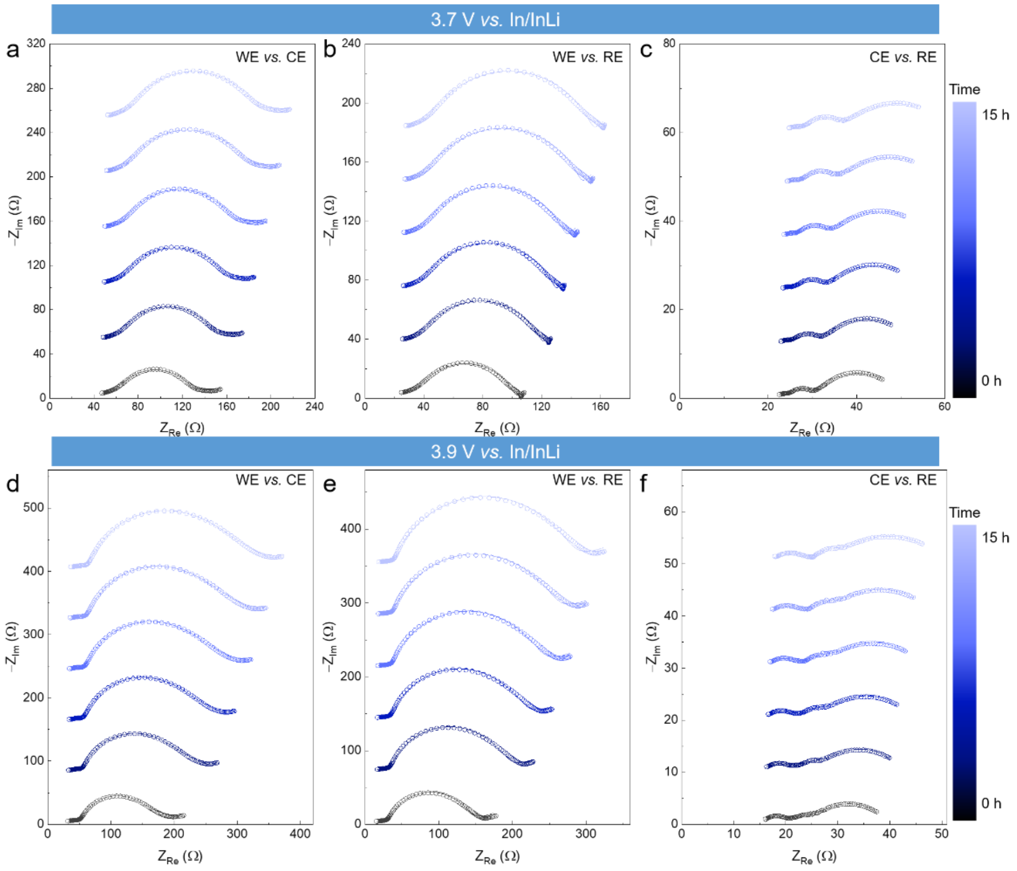

Supplementary Figure 5. EIS measurements of the 3-electrode cell during resting at 25 ˚C. The cell was charged to 3.7 V (a-c) and 3.9 V *vs.* In/InLi (d-f). (a and d), (b and e) as well as (c and f) show the impedance spectra and their corresponding fitting spectra of working electrode (WE) *vs.* counter electrode (CE), WE *vs.* reference electrode (RE) and CE *vs.* RE, respectively. Compared to the anode|SE interfacial resistance (*i.e.*, CE *vs.* RE), the resistance at the SE|cathode interface (*i.e.*, WE *vs.* RE) increases dramatically. This result indicates that the contribution of cathode interface dominates the overall impedance. It should be noted that the introduction of RE may influence the resistance due to the thicker separator and the geometry of RE.[1-2]

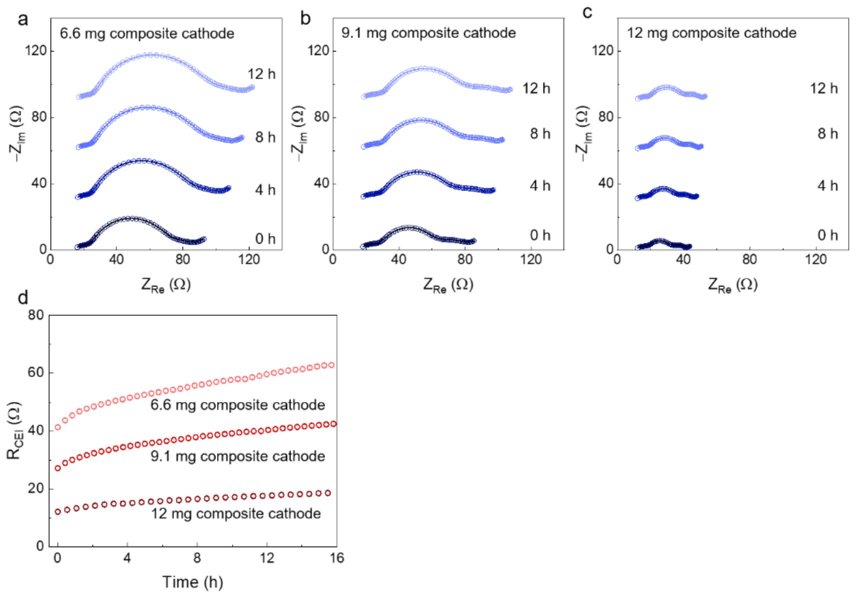

Supplementary Figure 6. Comparison of resistance growth in three cells with different mass loading. (a-c) Nyquist plots of three cells after charging up to 3.5 V *vs.* In/InLi. (d) R_{CEI} evolution with resting time.

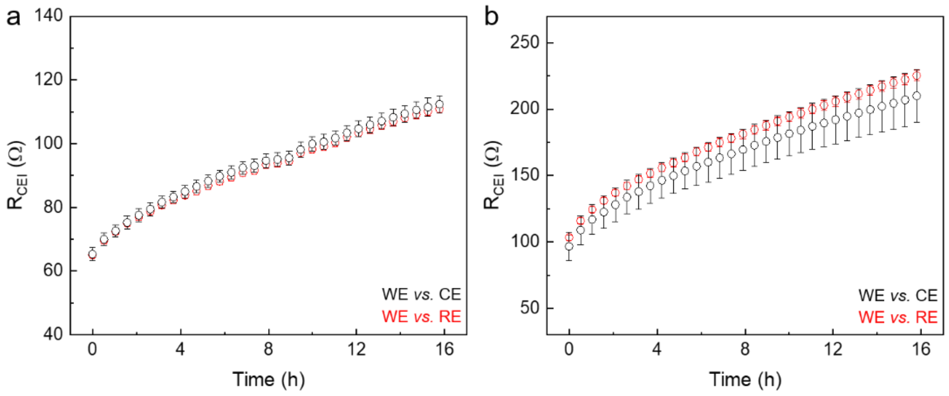

Supplementary Figure 7. Comparison of the fitting results of R_{CEI} based on the WE *vs.* CE and WE *vs.* RE impedance spectra. (a-b) show the fitting results of cells after charging to 3.7 V and 3.9 V *vs.* In/InLi, respectively. The error bars were marked to show the deviation due to the fitting process.

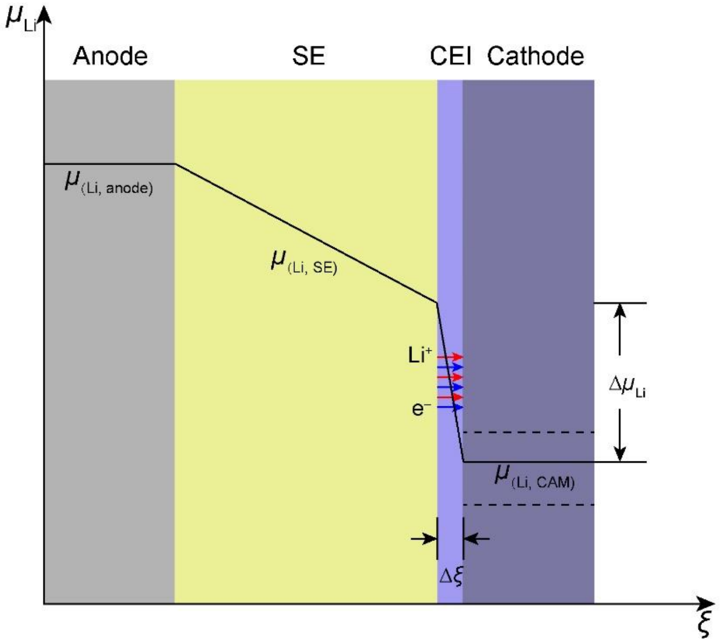

Supplementary Figure 8. Schematic diagram of lithium chemical potential in a full cell. The chemical potential difference serves as the driving force of the interfacial degradation (*i.e.* self-discharge), which leads to the growth of the degradation layer (CEI).

As shown in Supplementary Fig. 8, the diffusion-controlled degradation can be described with a Wagner-type model.[3] The assumption is that the growth of the product layer (the CEI) requires the coupled migration of ionic and electronic charge carriers. The growth rate is controlled by the charge carrier that exhibits the smaller transport coefficient (i.e., the electron in this case).

The chemical potential gradient of the neutral component Li drives the flux of Li across the CEI. Assuming quasi-stationary conditions in case of a slowly growing layer, we can apply Fick's first law, and the flux j_{Li^+} of Li^+ ions and j_{e^-} of electrons e^- can be written as:

$$j_{\mathrm{Li}^+} = -L_{\mathrm{Li}^+} \cdot \nabla\tilde{\mu}_{\mathrm{Li}^+} \tag{1}$$

$$j_{\mathrm{e}^-} = -L_{\mathrm{e}^-} \cdot \nabla\tilde{\mu}_{\mathrm{e}^-} \tag{2}$$

The L_i and $\nabla\tilde{\mu}_i$ denote the phenomenological transport coefficients and gradients of the electrochemical potential of ions and electrons, respectively. Since electroneutrality is maintained, the flux of neutral Li results as:

$$j_{\mathrm{Li}} = j_{\mathrm{Li}^+} = j_{\mathrm{e}^-} = -\frac{L_{\mathrm{Li}^+} \cdot L_{\mathrm{e}^-}}{L_{\mathrm{Li}^+} + L_{\mathrm{e}^-}} \cdot (\nabla\mu_{\mathrm{Li}^+} + \nabla\mu_{\mathrm{e}^-}) = -\frac{L_{\mathrm{Li}^+} \cdot L_{\mathrm{e}^-}}{L_{\mathrm{Li}^+} + L_{\mathrm{e}^-}} \cdot \nabla\mu_{\mathrm{Li}} \tag{3}$$

The conductivity σ_i of the component i is related to its L_i by

$$\sigma_i = (z_i \mathrm{F})^2 \cdot L_{\mathrm{i}} \tag{4}$$

with Faraday's constant F and the charge number z_i. Eq. 3 can then be written as:

$$j_{\mathrm{Li}} = -\frac{1}{F^2} \cdot \frac{\sigma_{\mathrm{Li}^+} \cdot \sigma_{\mathrm{e}^-}}{\sigma_{\mathrm{Li}^+} + \sigma_{\mathrm{e}^-}} \cdot \nabla\mu_{\mathrm{Li}} = -\frac{1}{F^2} \cdot \frac{\sigma_{\mathrm{Li}^+} \cdot \sigma_{\mathrm{e}^-}}{\sigma_{\mathrm{Li}^+} + \sigma_{\mathrm{e}^-}} \cdot \frac{d\mu_{\mathrm{Li}}}{d\xi} \tag{5}$$

The flux of Li along the CEI with its thickness ξ across the growing CEI leads to further growth.

$$j_{\mathrm{Li}} = \frac{1}{S} \cdot \frac{dn_{\mathrm{Li}}}{dt} = \frac{x}{S} \cdot \frac{dV}{V_{\mathrm{m}} \cdot dt} = \frac{x\, d\xi}{V_{\mathrm{m}} \cdot dt} \tag{6}$$

Here, S denotes the crossed surface area, n_{Li} represents the molar amount of neutral Li, x denotes the number of Li atoms extracted from the CEI per V_{m}, V_{m} represents the average molar volume of the CEI per Li atom, V represents the volume of the CEI layer. By combining Eq. 5 and 6, a further integration process leads to Eq. 7 and 8.

$$(d\xi)^2 = [-\frac{V_{\mathrm{m}}}{x\, F^2} \cdot \frac{\sigma_{\mathrm{Li}^+} \cdot \sigma_{\mathrm{e}^-}}{\sigma_{\mathrm{Li}^+} + \sigma_{\mathrm{e}^-}} \cdot d\mu_{\mathrm{Li}}]dt \tag{7}$$

$$(\Delta\xi)^2 = \left[-\frac{V_{\mathrm{m}}}{\bar{x}\, F^2} \cdot \frac{\overline{\sigma_{\mathrm{Li}^+}} \cdot \overline{\sigma_{\mathrm{e}^-}}}{\overline{\sigma_{\mathrm{Li}^+}} + \overline{\sigma_{\mathrm{e}^-}}} \cdot \int_{\mu_{(\mathrm{Li,SE})}}^{\mu_{(\mathrm{Li,CAM})}} d\mu\right] t \tag{8}$$

$$(\Delta\xi)^2 = \left[-\frac{V_{\mathrm{m}}}{\bar{x}\, F^2} \cdot \frac{\overline{\sigma_{\mathrm{Li}^+}} \cdot \overline{\sigma_{\mathrm{e}^-}}}{\overline{\sigma_{\mathrm{Li}^+}} + \overline{\sigma_{\mathrm{e}^-}}} \cdot \Delta\mu_{\mathrm{Li}}\right] t \tag{9}$$

In Eq.8, the averaged partial conductivities of ions and electrons across the CEI are introduced, in order to simplify the integral of the driving force. The thickness of the degradation layer could be estimated to be:

$$\Delta\xi = \sqrt{\frac{V_{\mathrm{m}}}{x\, F^2} \cdot \frac{\overline{\sigma_{\mathrm{Li}^+}} \cdot \overline{\sigma_{\mathrm{e}^-}}}{\overline{\sigma_{\mathrm{Li}^+}} + \overline{\sigma_{\mathrm{e}^-}}} \cdot \Delta\mu_{\mathrm{Li}}} \cdot \sqrt{t} \tag{9}$$

Therefore, the interfacial resistance – caused by the CEI - can be calculated from the thickness divided by surface area (S) and average conductivity of the CEI layer ($\overline{\sigma_{\mathrm{CEI}}}$).

$$R_{\mathrm{CEI}} = \frac{1}{S\overline{\sigma_{\mathrm{CEI}}}} \sqrt{\frac{V_{\mathrm{m}}}{x\, F^2} \cdot \frac{\overline{\sigma_{\mathrm{Li}^+}} \cdot \overline{\sigma_{\mathrm{e}^-}}}{\overline{\sigma_{\mathrm{Li}^+}} + \overline{\sigma_{\mathrm{e}^-}}} \cdot \Delta\mu_{\mathrm{Li}}} \cdot \sqrt{t} = \frac{1}{S\overline{\sigma_{\mathrm{CEI}}}} \cdot k\sqrt{t} = k'\sqrt{t} \tag{10}$$

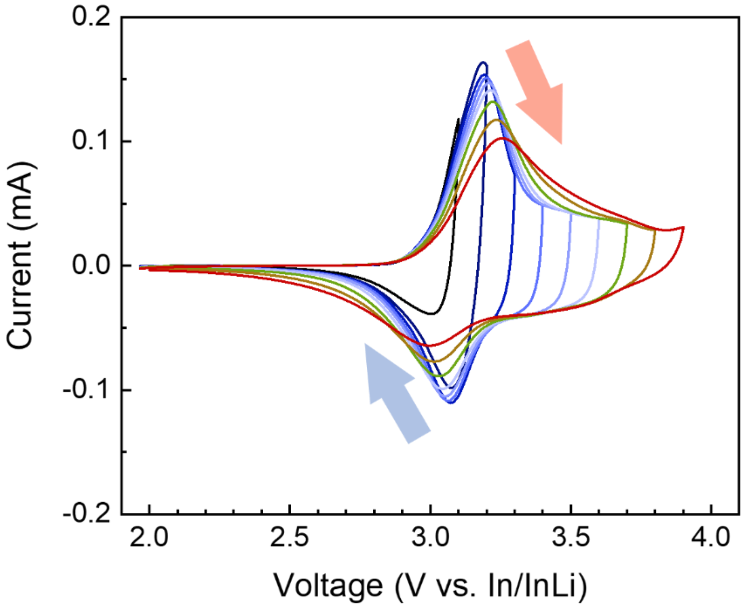

Supplementary Figure 9. Stepwise cyclic voltammetry of a In/InLi|LGPS|NCM622 cell at 25 °C. The cell was set with gradually increasing the positive cutoff voltage in steps of 0.1 V. The scan rate was 0.01 mV s^{-1}.

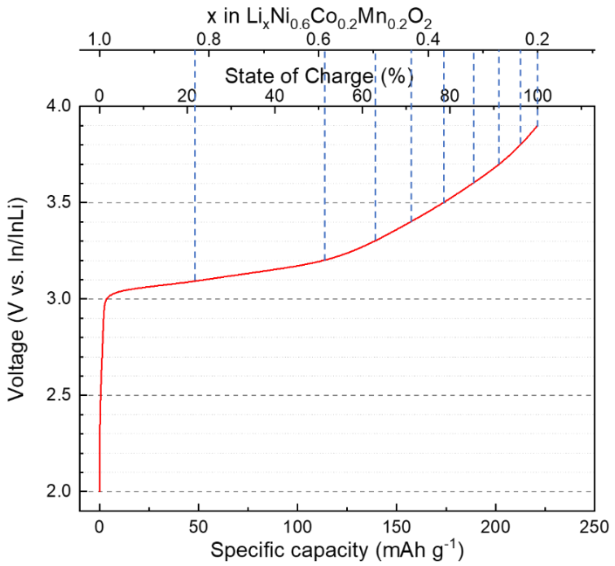

Supplementary Figure 10. The correlation between voltage against specific capacity, SOC and Li concentration in NCM622 cathode material. The SOC is defined as 100% when the voltage reaches 3.9 V *vs*. In/InLi.

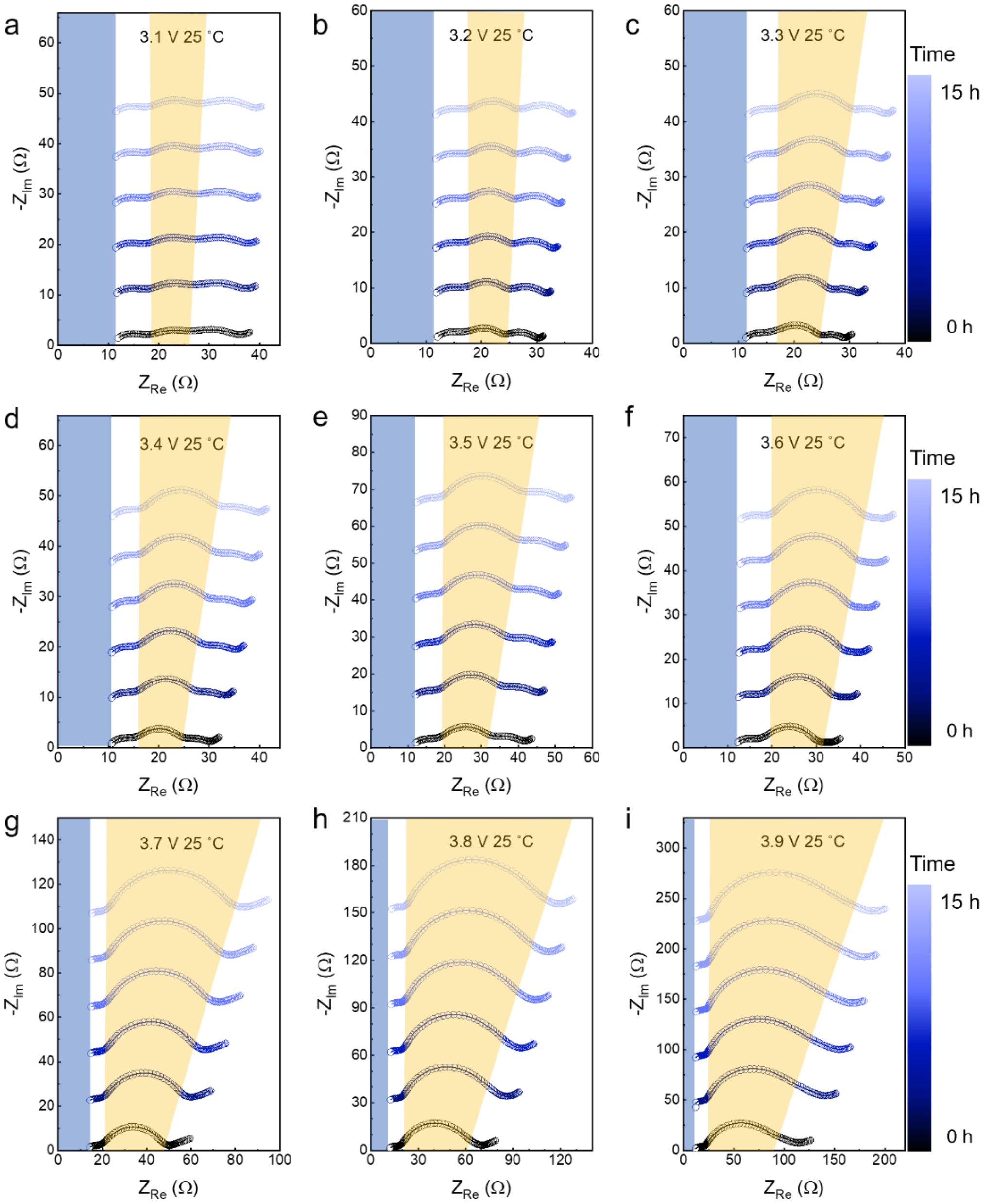

Supplementary Figure 11. Impedance changes with time at different potentials at 25 ˚C. Electrochemical impedance spectra were collected after charging to (a) 3.1 V, (b) 3.2 V, (c) 3.3 V, (d) 3.4 V, (e) 3.5 V, (f) 3.6 V, (g) 3.7 V (h) 3.8 V and (i) 3.9 V *vs.* In/InLi. The impedance contribution of the cathode/electrolyte interface is marked by yellow background.

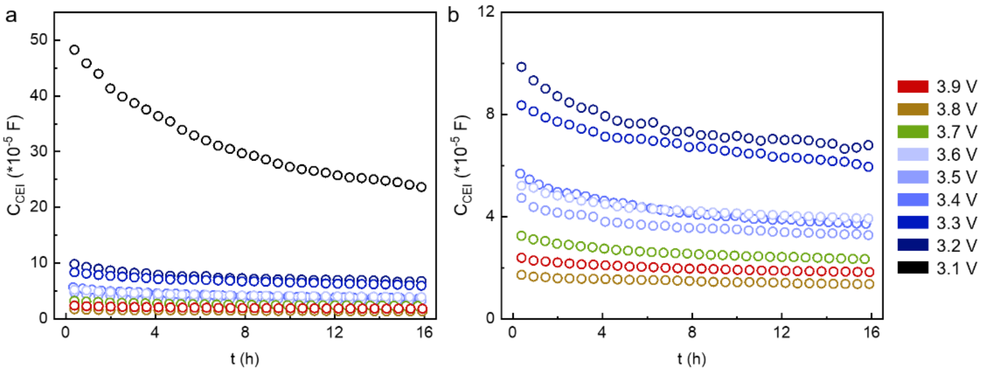

Supplementary Figure 12. (a) Change of the CEI capacitance with time at different SOC at 25 ˚C. (b) The enlarged plot shows more details of graph (a).

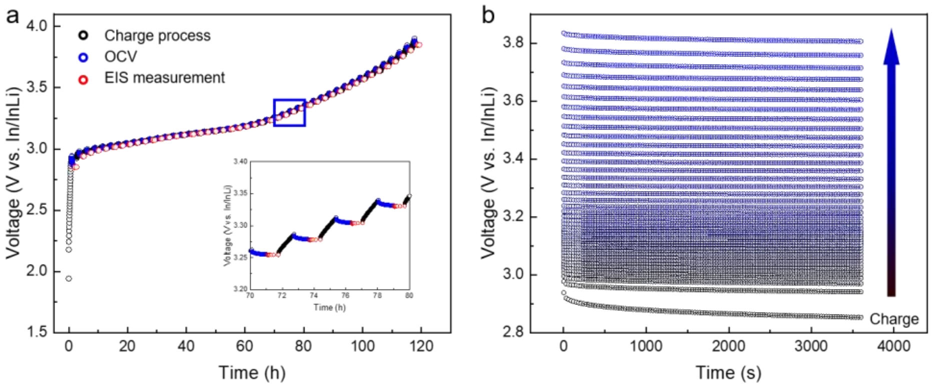

Supplementary Figure 13. (a) Example of a galvanostatic intermittent titration experiment and (b) the corresponding voltage-time curves during relaxation.

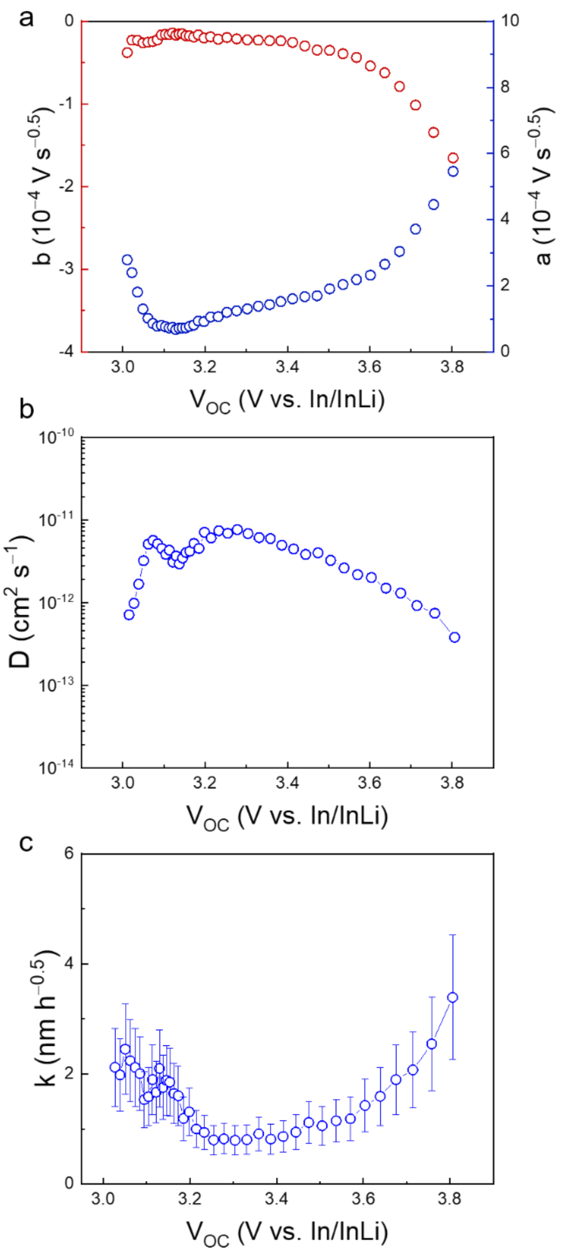

Supplementary Figure 14. (a) The coefficients *a* and *b*, and (b) the corresponding lithium diffusion coefficient, and (c) degradation rate constant determined from the relaxation curves as function of voltage, i.e., as a function of SOC.

To estimate the growth rate of the degradation layer, the changes in the molar volume are neglected and thereby the molar volume of LGPS (283.5 cm^3 mol^{-1}) is applied.[4] The molar volume based on Li atom can be calculated to be:

$$V_{\mathrm{m}}(\mathrm{Li}) = \frac{V_{\mathrm{m}}(\mathrm{LGPS})}{10} = 28.4\ \mathrm{cm}^3\ \mathrm{mol}^{-1}$$

Based on the rough estimation, the growth rate was semi-quantified under the assumption that the average molar volume is in the range of 20 - 40 cm^3 mol^{-1}.

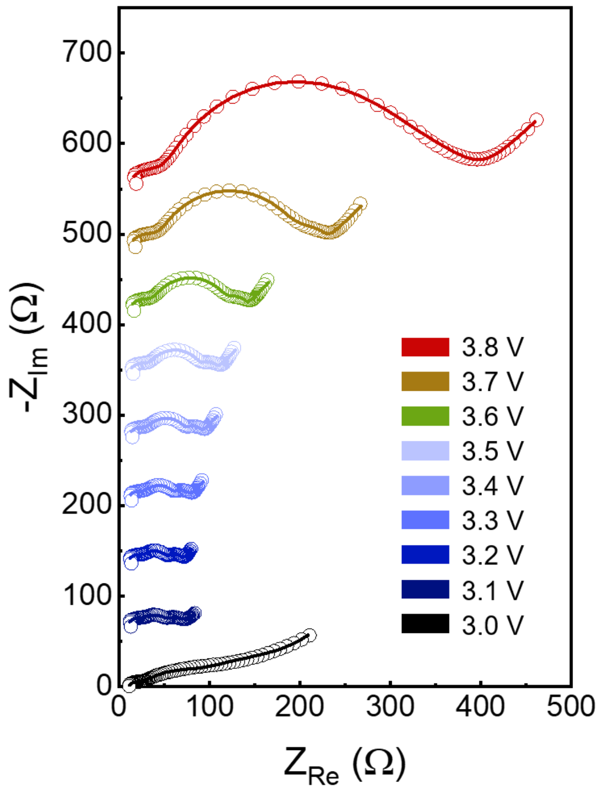

Supplementary Figure 15. Nyquist plots of the impedance data with different V_{OC} at 25˚C. The impedance measurements were conducted during GITT measurements.

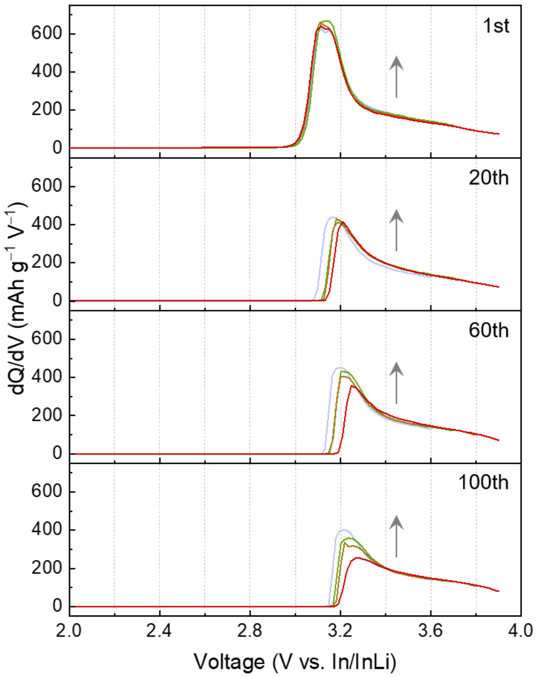

Supplementary Figure 16. The *dQ/dV* curves of four cells with different cutoff voltages (Light blue: 2-3.6 V, green: 2-3.7 V, yellow: 2-3.8 V, red: 2-3.9 V).

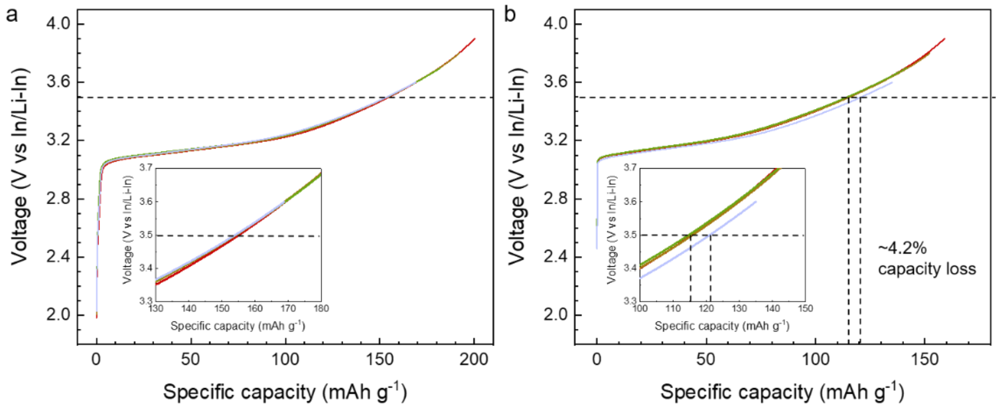

Supplementary Figure 17. Galvanostatic charge curves at (a) 1st and (b) 2nd cycle with different upper cutoff voltages. The capacity at 3.5 V (dash lines) is marked to evaluate the fraction of active materials. Compared with SSB charged up to 3.6 V (light blue line), the SSBs charged up to 3.7-3.9 V (yellow, green and red lines) exhibit 4.2% capacity loss. Insets show the enlarged charge curves.

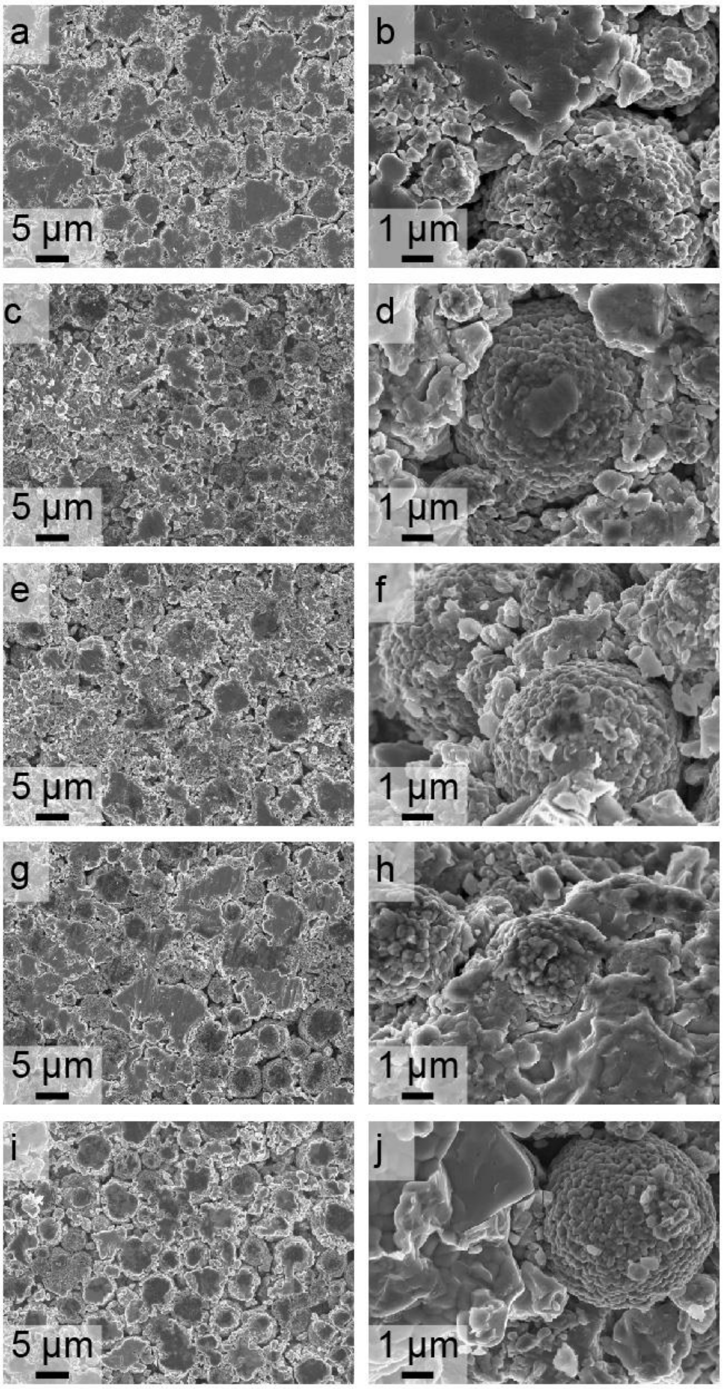

Supplementary Figure 18. SEM images of the composite cathodes at (a, b) OCV stage and after charged up to (c, d) 3.6 V, (e, f) 3.7 V, (g, h) 3.8 V and (i, j) 3.9 V.

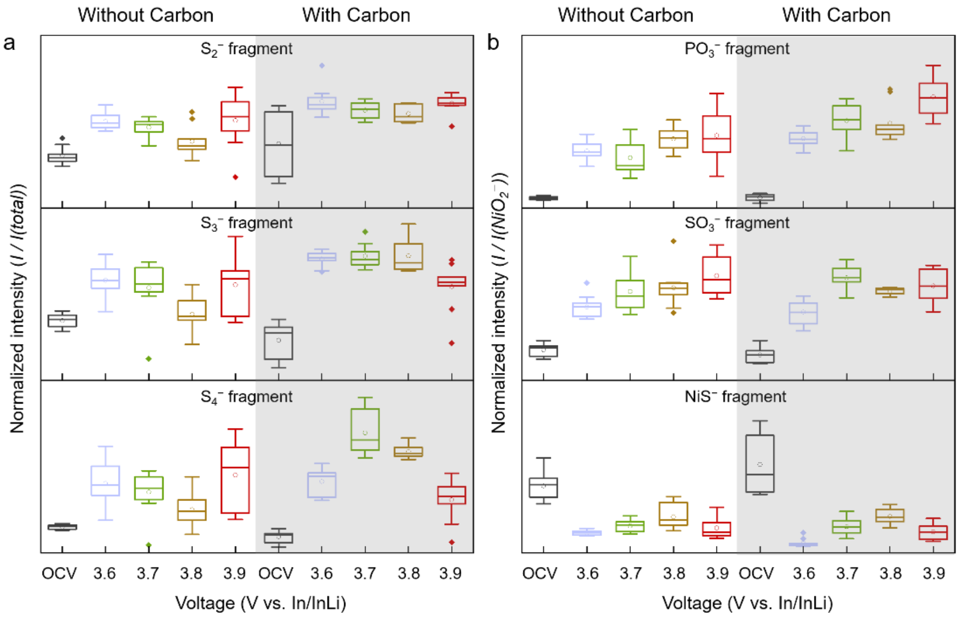

Supplementary Figure 19. Comparison of the normalized intensities of (a) S_2^-/S_3^-/S_4^- and (b) PO_3^-/SO_3^-/NiS^- fragments of cathode pellets with/without carbon additive at different SOCs. The intensities of S_2^-/S_3^-/S_4^- signals were normalized with the total ion intensity, and the intensities of PO_3^-/SO_3^-/NiS^- fragments were normalized in relation to the NiO_2^- signal.

Supplementary Table 1. Slopes of linear fits of R_{CEI} with different cutoff voltage at 25 ˚C.

Cutoff voltage (V *vs.* In/InLi)	3.1	3.2	3.3	3.4	3.5	3.6	3.7	3.8	3.9
Slope of the linear fit ($\Omega \cdot h^{-0.5}$)	0.59	0.60	1.22	1.49	1.91	2.24	6.58	10.11	15.35

Supplementary Table 2. Fitting results of the temperature-dependent impedance measurements shown in Figure 6 (see main text).

Cutoff voltage (V)	Parameters	10 ˚C	25 ˚C	40 ˚C
3.6 V	Rate constant ($\Omega\ h^{-0.5}$)	7.36	2.24	3.57
	Charge transfer (Ω)	80.60	10.13	10.50
	Normalized rate constant ($h^{-0.5}$)	0.09	0.22	0.34
	Activation energy (eV)		0.352± 0.090	
3.7 V	Rate constant ($\Omega\ h^{-0.5}$)	12.35	6.58	5.32
	Charge transfer (Ω)	80.60	21.95	10.24
	Normalized rate constant ($h^{-0.5}$)	0.15	0.30	0.52
	Activation energy (eV)		0.325± 0.003	
3.8 V	Rate constant ($\Omega\ h^{-0.5}$)	14.43	10.11	9.89
	Charge transfer (Ω)	124.89	22.00	15.74
	Normalized rate constant ($h^{-0.5}$)	0.12	0.46	0.63
	Activation energy (eV)		0.437± 0.085	
3.9 V	Rate constant ($\Omega\ h^{-0.5}$)	21.31	15.35	20.34
	Charge transfer (Ω)	175.19	50.56	12.88
	Normalized rate constant ($h^{-0.5}$)	0.12	0.30	1.58
	Activation energy (eV)		0.649± 0.127	

References:

1. Epding, B., Broda, A., Rumberg, B., Jahnke, H. & Kwade, A. Development of durable 3-electrode lithium-ion pouch cells with LTO reference mesh: aging and performance studies. *J. Electrochem. Soc.* **166**, A1550-A1557 (2019).
2. Adler, S. B. Reference electrode placement in thin solid electrolytes. *J. Electrochem. Soc.* **149**, E166 (2002).
3. Schmalzried, H. *Chemical kinetics of solids.*(VCH, 1995).
4. Dewald, G. F., et al. Experimental assessment of the practical oxidative stability of lithium thiophosphate solid electrolytes. *Chem. Mater.* **31**, 8328-8337 (2019).
